# Validation of temporal parameters within the skating sub-techniques when roller skiing on a treadmill, using inertial measurement units

**DOI:** 10.1371/journal.pone.0270331

**Published:** 2022-08-18

**Authors:** Frédéric Meyer, Trine M. Seeberg, Jan Kocbach, Jørgen Danielsen, Øyvind Sandbakk, Andreas Austeng

**Affiliations:** 1 Department of Informatics, Digital Signal Processing Group, University of Oslo, Oslo, Norway; 2 SINTEF Digital, Smart Sensor Systems, Oslo, Norway; 3 Department of Neuromedicine and Movement Science, Centre for Elite Sports Research, Norwegian University of Science and Technology, Trondheim, Norway; Universita degli Studi di Verona, ITALY

## Abstract

The aim of this study was to develop and validate a method using inertial measurements units (IMUs) to determine inner-cycle parameters (e.g., cycle, poles and skis contact, and swing time) and the main sub-techniques (i.e., G2, G3 and G4) in cross-country roller ski skating on a treadmill. The developed method is based on the detection of poles and skis initial and final contacts with the ground during the cyclic movements. Thirteen well-trained athletes skied at different combinations of speed (6–24 km∙h^-1^) and incline (2–14%) on a treadmill using the three different sub-techniques. They were equipped with IMUs attached to their wrists and skis. Their movements were tracked using reflective markers and a multiple camera infrared system. The IMU-based method was able to detect more than 99% of the temporal events. It calculated the inner-cycle temporal parameters with a precision ranging from 19 to 66 ms, corresponding to 3.0% to 7.8% of the corresponding inner-cycle duration. The obtained precision would likely allow differentiation of skiers on different performance levels and detection of technique changes due to fatigue. Overall, this laboratory validation provides interesting possibilities also for outdoor applications.

## Introduction

Most endurance sports can be described as a succession of cycles performed in a particular motion [1]. In such cases, an accurate detection of the start and the end of each cycle is a prerequisite for precise comparison of multiple occurrences of the same pattern. An accurate detection of each cycle will consequently allow for the determination of spatio-temporal parameters, which is regarded useful for coaches and athletes when analysing performance, for improving movement technique and efficiency, and possibly for reducing the risk of injury [2].

**Fig 1:**
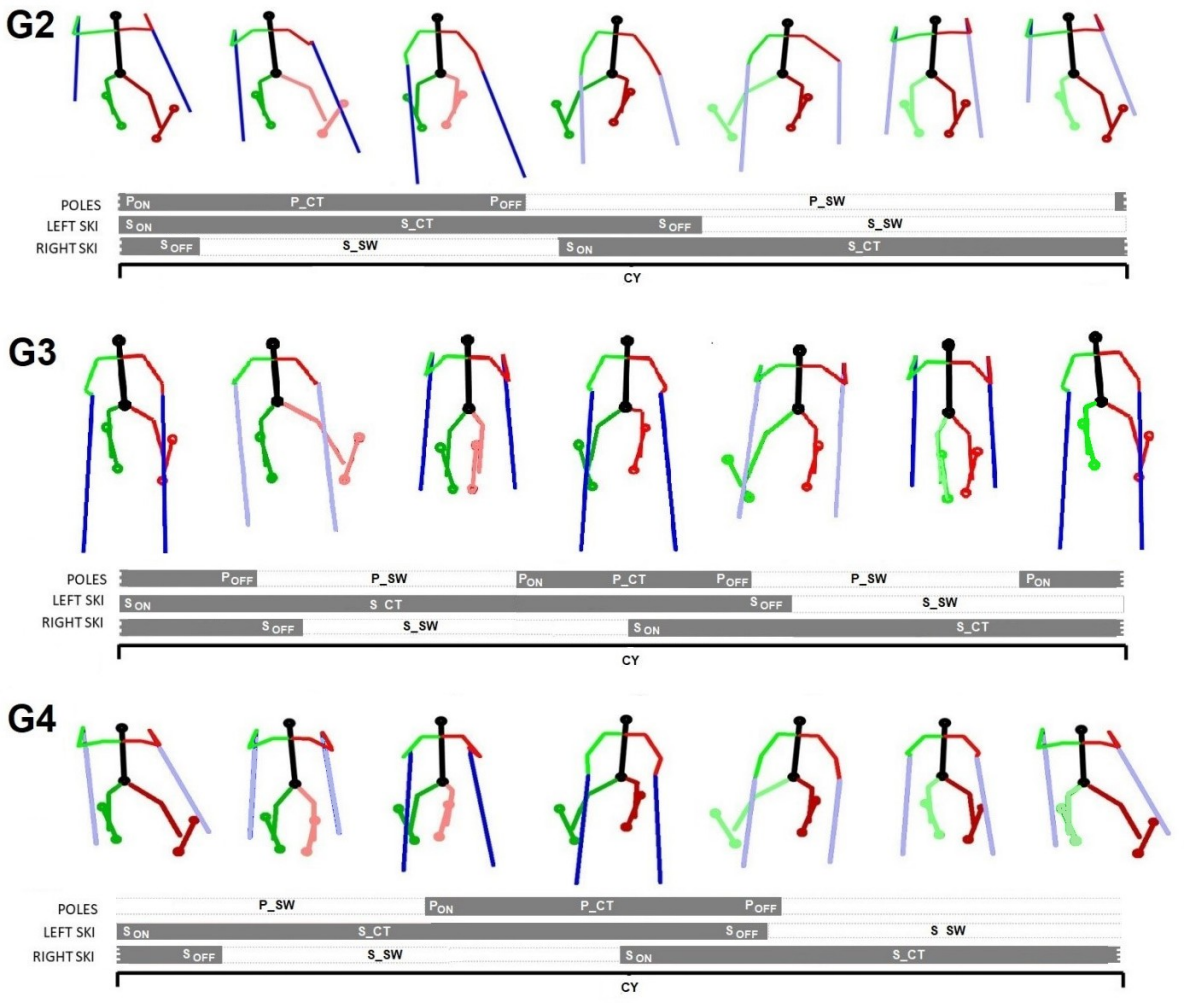
Representation of the three main skating sub-techniques (G2, G3 and G4), and their corresponding temporal events: Pole and ski initial contact (P_ON_ and S_ON_), final contact (P_OFF_ and S_OFF_); and inner-cycle phases: Pole and ski contact time (P_CT and S_CT) and swing time (P_SW and S_SW), as well as cycle time (S_CY). The dark colour of legs and poles illustrate the contact with the ground.

In cross-country skiing, athletes employ multiple sub-techniques to ski as efficient as possible across varying terrain, both in the classical and skating styles. These sub-techniques can be compared to gears in cycling, as the athlete chooses the sub-technique that is best adapted to external conditions [3–5]. The multiple sub-techniques in ski skating imply different coordination patterns of body segments to optimize concurrent propulsion from poles and skis within a cycle. Detection of the initial and final ground contact for poles and skis (P_ON,_ S_ON_, P_OFF_, S_OFF_) will allow determination of the associated temporal parameters, such as cycle duration (CY), pole and ski contact (P_CT and S_CT) and swing time (P_SW and S_SW), as well as the duration between temporal events and the type of sub-technique used (Fig 1). In previous studies, these parameters were mainly obtained by using a marker-based, multiple cameras system or force sensors [6, 7]. However, such complex systems are rarely used on snow, and only in an indoor tunnel, due to the complexity of the experimental setup and the need of specific equipment [8, 9]. In addition, complex and relatively heavy differential global navigation satellite systems have been used to detect cycles and sub-technique in the field [10].

A more easily available alternative to these complex systems is wearable sensors such as inertial measurement units (IMUs). These sensors are now widely used in sport science and have the advantages of being light, unobtrusive, and allow for long term recording. In addition, several devices can be worn simultaneously and synchronized to get data from different parts of the body and/or equipment. However, the challenges when using IMUs are first to find the best location for the sensors and second the need for developing a dedicated data analysis tool for each application that provides accurate results. Such a process has been used in walking [11], running [12], swimming [13], ski mountaineering [14], and classical cross-country skiing [15] to determine the accuracy of temporal event associated with each movement cycle. In the latter cross-country skiing publication, IMUs were attached to the poles and skis.

In the skating style, visual observation of the outcome of an IMU mounted on the upper back of the athlete [16] and then machine learning has been used on IMU data to determine cycles and the usage of sub-technique using a smartphone attached to the front of the chest [17] or four IMUs placed on the wrists and on the skis [18]. A method based on attractors [19] was applied on IMUs attached to the wrists and ankles to analyse technique differences among athletes [20]. Finally, biomechanical analysis of the different skating sub-techniques has been performed by use of IMU data [21], in which a basic validation of the P_ON_ S_ON_, P_OFF_, S_OFF_ was performed. These temporal events were determined using IMUs placed on the ski boots and poles and compared with approximately 500 events detected manually from 50 Hz video. The authors highlighted the difficulty to accurately determine the S_ON_ manually. Accordingly, there is clearly a need for a robust and easy-to-use method for detecting temporal events in ski skating sub-techniques, validated with an accurate reference system over many participants and many slope/speed combinations. A natural step is to first develop a method in a laboratory setting, that provides a solid basis for further adaptation to an outdoor solution.

The aim of the present study was therefore to develop and validate a method using distributed IMUs to determine inner-cycle parameters (e.g. cycle, poles and skis contact and swing time) and the performed sub-technique based on the detection of temporal events for poles and skis within the three main skating sub-techniques while roller skiing on a treadmill.

## Methods

### Participants

Thirteen well-trained male athletes, consisting of eight cross-country skiers (International Ski Federation distance points: 47.3 ± 20.9), and five national level biathletes participated in this study. The participant characteristics were as follows: ages of 24.8 ± 2.7 years, body heights of 184 ± 6 cm, body masses of 79.3 ± 5.2 kg, and peak oxygen uptakes of 69 ± 3.7 mL·min^-1^·kg^-1^. The Regional Committee for Medical and Health Research Ethics waives the requirement for ethical approval for such studies. Therefore, the study was done in accordance with the institutional requirements and in line with the Helsinki declaration. Approval for data security and handling was obtained from the Norwegian Center for Research Data before the study. Prior to the data collection, all skiers provided written informed consent to voluntarily take part in the study. The skiers were informed that they could withdraw from the study at any point in time without providing a reason for doing so.

### Equipment

The protocol was performed on a 3-by-5-m motor-driven roller ski treadmill (Forcelink S-mill, Motekforce Link, Amsterdam, The Netherlands). The skier’s used poles of their individually chosen lengths of 90 ± 2% of body height. All skiers wore their own skating boots but used the same pair of skate elite roller skis (IDT Sports, Lena, Norway) with an NNN binding system (Rottefella, Klokkarstua, Norway). Slope and speed of the treadmill were calibrated before and after the study using the Qualisys system and Qualisys Track Manager software (Qualisys AB, Gothenburg, Sweden). Participants wore a safety harness connected to an automatic emergency brake during the high-intensity parts of the tests.

**Fig 2:**
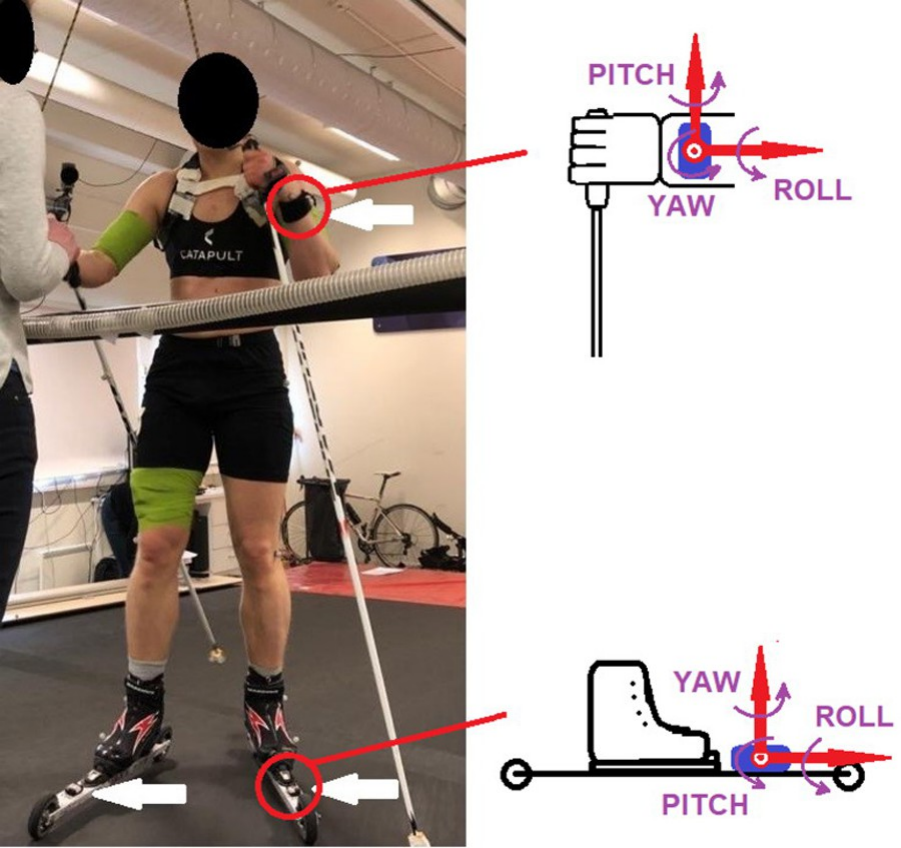
Experimental setup, showing the treadmill, the inertial measurement units (IMUs) attached to the wrist and to the skis (white arrows), the reflective markers, and a camera in the background. Detailed axis definition for the IMUs is also provided.

Motion data were collected using two different systems. First, a multiple camera system consisting of eight Oqus 400 infrared cameras (Qualysis AB, Gothenburg, Sweden), capturing three-dimensional position characteristics of passive reflective markers at 200 Hz, was used as reference. Markers were placed on the lateral sides of the carbide pole tips and on the lateral sides of the poles, 10 cm below the pole handles, as well as two markers at the front and back of each ski. Second, four IMUs (Physilog 5, GaitUpSA, Lausanne, Switzerland), 11 grams each and composed of a 3D accelerometer (range of ±16G) and a 3D gyroscope (range ±2000°/s) with a sampling frequency of 256 Hz were used to determine temporal events. Before starting the test, the IMUs were mounted using Velcro straps on the left and right wrists, and in front of the binding on both skis (Fig 2). The wrists and skis were chosen based on previous studies [22, 23] as a good option to gather good data and for commercial device location (e.g., to include such sensors in smart watches and pods). Synchronization between IMUs was performed internally using a proprietary procedure based on radio signal, and the synchronization between the IMUs and the multiple camera system was performed manually, using a dedicated pole plant at the beginning of each trial.

### Procedures

The protocol consisted of two consecutive testing days. Day 1 involved a 5-min warm-up, twelve submaximal exercise bouts of 4 minutes at constant speed, followed by a maximal incremental test. The twelve submaximal bouts consisted of three different sub-techniques (i.e., G2, G3 and G4) at four different intensities, performed in randomized order (G2: 12% incline at 6/7/8/9 km∙h^-1^, G3: 5% incline at 10/12/14/16 km∙h^-1^and G4: 2% incline at 15/18/21/24 km∙h^-1^). A minimum of 2 minutes of recovery was given between each condition. For the maximal incremental test, the starting incline and speed were 10.5% and 11 km∙h^-1^_,_ respectively. The speed was then kept constant, while the incline was subsequently increased by 1.5% every minute until 14.0%. Thereafter, the speed was increased by 1 km∙h^-1^ every minute until exhaustion. Athletes were free to choose and adapt their sub-technique during the incremental test.

Day 2 consisted of a warm-up of 13 minutes before two stages of 21 minutes of a) low- and b) high-intensity with freely chosen sub-technique across a simulated terrain profile on the treadmill. The track was organized as seven identical 3-min laps consisting of four different segments simulating a moderate uphill (5% incline), a flat segment (2% incline), a steep uphill (12% incline) and a simulated downhill (where the athlete was holding a rope in a tack position). The profile of the track was designed according to standards of the International Ski Federation, where the standard sub-techniques could naturally be utilized. The high-intensity stage was immediately followed by an incremental all-out test with gradually increasing speed every 15-seconds until exhaustion. The slopes and speeds used were the same for all the athletes, and based on pilot testing and on previous research, representing typical slopes where these techniques are employed [5, 24]. The present study is part of a larger data collection, and more details are provided in Seeberg et al. [25].

### Data processing

Data from each trial were processed using a dedicated Matlab procedure (Matlab R2019a, The MathWorks Inc., Natick, Massachusetts, USA). Skiing cycles were defined as proposed previously [26], starting when the left ski hit the ground. For the reference system, P_ON_, P_OFF_, S_ON_ and S_OFF_ were determined by first preselecting sections were the poles and skis were close to the treadmill, and then using the maximum of the second derivative of the vertical position of each pole and each ski. The following temporal parameters were then calculated P_CT, P_SW, S_CT, S_SW, S_CY, delay between P_ON_ and S_ON_ (defined as the absolute value of the shortest time difference between P_ON_ and S_ON_), and delay between S_OFF_ and P_OFF_.

**Fig 3:**
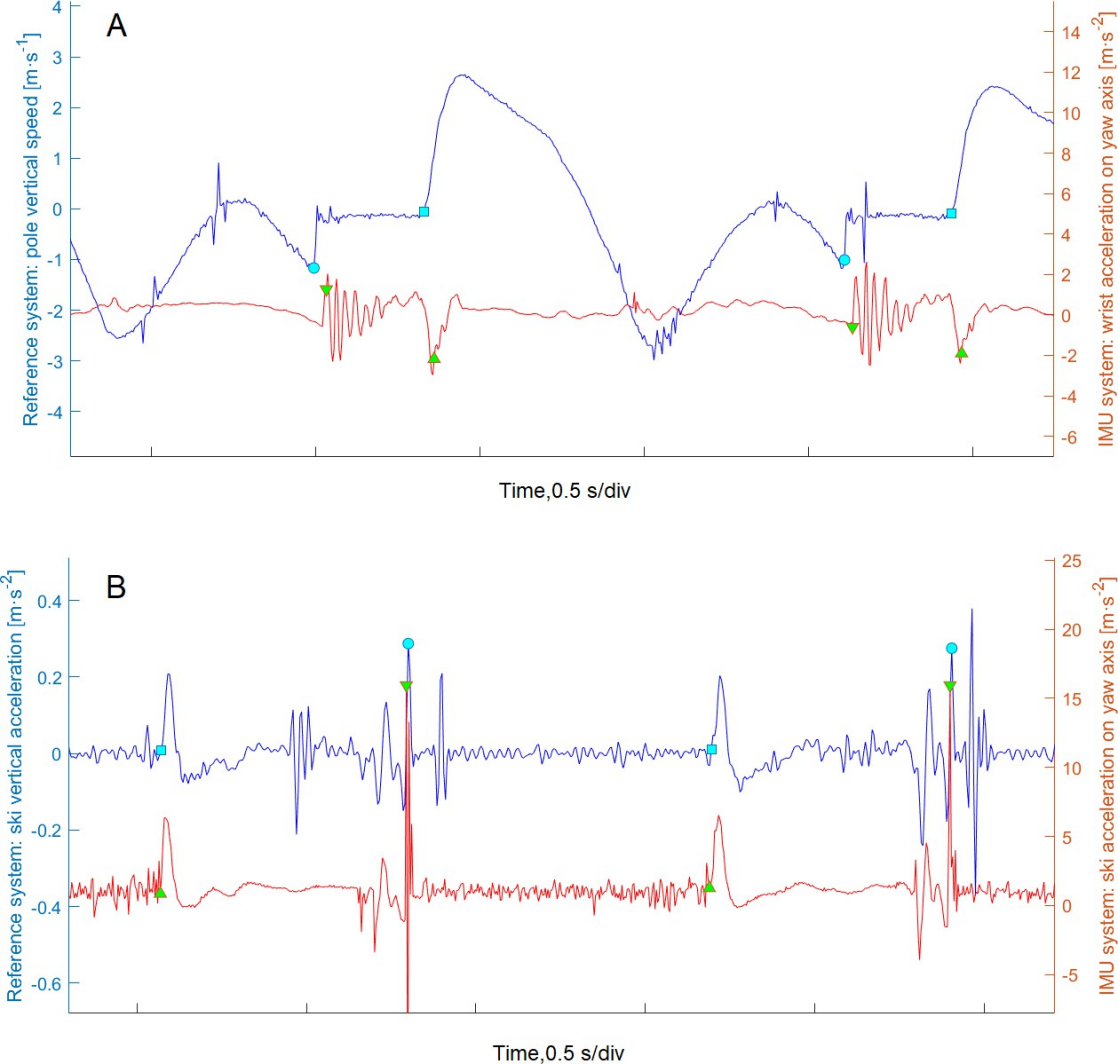
Pole (**A**) and ski (**B**) event detection both from the reference system and from inertial measurement units (IMU) for the G3 sub-technique. ● and ■ indicates initial and final contact for the reference system, ▼ and ▲ indicates initial and final contact for the IMU system.

For the two IMUs placed on the wrists (Fig 2), the angular velocity around the yaw axis of the wrist was first integrated, and a 2^nd^ order 0.1 Hz high pass Butterworth filter was then applied on the signal to remove the drift and determine the overall motion frequency of the arm’s cyclic movements. All the filter configurations were chosen empirically to obtain the expected effect on a randomly chosen selection of three trials. Each cycle was then processed to determine the P_ON_ and P_OFF._ To determine P_ON_, the local maximum of the yaw angle of the wrist was first detected. Starting from that point, the first positive acceleration peak with a prominence higher than 1 m·s^-2^ was detected on the yaw axis of the wrist IMU. Finally, P_ON_ was set as the last negative acceleration peak before the previously determined positive acceleration (Fig 3A). For P_OFF_, the yaw acceleration of the wrist IMU was first filtered with a 2^nd^ order 5 Hz high pass Butterworth filter to remove slow components of the motion. The P_OFF_ was then set as the lowest acceleration peak of the filtered signal, close to the minimum yaw angle of the wrist. This lowest peak reflects the change from backward to forward arm swing, preparing for the next cycle.

For the two IMUs placed on the skis (Fig 2), a 2^nd^ order 2 Hz low pass Butterworth filter was first applied on the roll angular velocity (around the longitudinal axis of the skis) to determine the overall motion frequency of the leg cyclic movement. Each cycle was then processed to determine the S_ON_ and S_OFF_. The S_ON_ was obtained by first integrating the roll angular velocity and then applying a 2^nd^ order 0.1 Hz high pass Butterworth filter to remove the drift. A first approximation of S_ON_ was set as the local maximum of the filtered signal. The precise S_ON_ was determined as the maximal acceleration peak of the vertical component (yaw axis) on the raw signal (Fig 3B). Finally, the S_OFF_ was then obtained by first determining the minimum roll angle of the skis at the end of the cycle, and then finding the highest negative acceleration peak of the vertical component (yaw axis) on the raw signal.

### Data analysis

The temporal events were independently determined with both the reference system and the IMUs. Each event found with the IMUs was attributed to the closest event found by the reference system. The non-attributed events on the reference system were considered as missed events for the IMUs. Then, the time difference between the two methods was analysed, and the different temporal parameters were calculated. Finally, these parameters were compared, both in absolute and relative terms. For each parameter, the bias (intra-trial mean) and the precision (intra-trial standard deviation) were calculated for all cycles within a trial. The results from all trials were then combined to determine the overall median and interquartile range (IQR) for both the bias and the precision, resulting in four inter-trial statistics: The median bias (b_μ_), the IQR of the bias (b_σ_), the median precision (σ_μ_) and the IQR of the precision (σ_σ_) [12]. The median and IQR were used to describe the inter-trial statistics as the intra-trial bias and precision were not normally distributed.

The precision of the different sub-techniques was compared with a Kruskal Wallis non-parametric One-way ANOVA, as the normality of the distribution was not respected. In case of significant main effect, a Dwass-Steel-Critchlow-Fligner pairwise comparison post-hoc test was performed to identify the different sub-techniques. The alpha level was set at 0.05.

**Fig 4:**
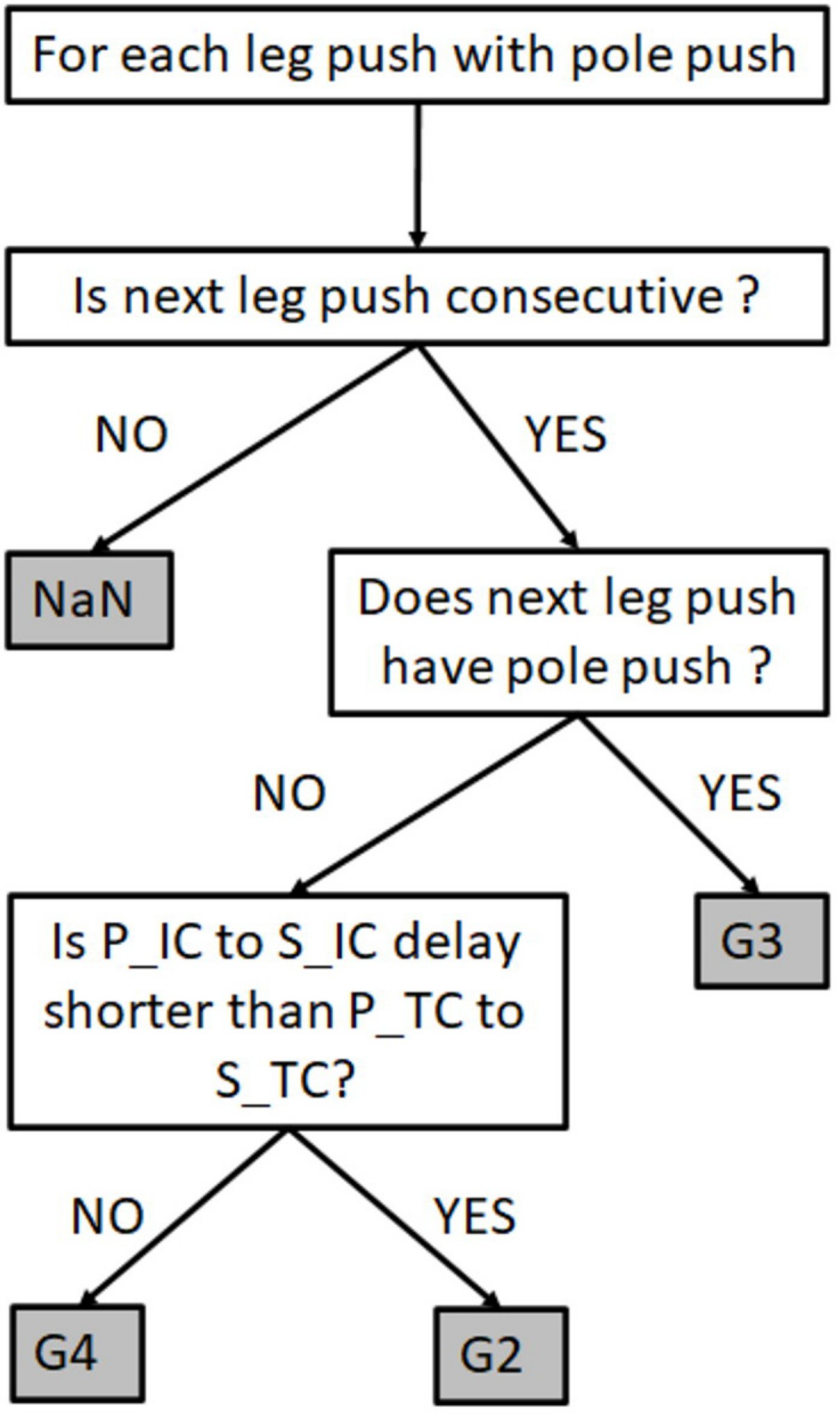
Decision-tree for sub-technique (G2, G3 and G4) determination. NaN means undetermined category, P_ON_ is the pole initial contact, P_OFF_ is the pole final contact, S_ON_ is the ski initial contact and S_OFF_ is the ski final contact.

Based on a visual observation of the videos, a simple decision tree was used to determine the sub-techniques on both the reference and the IMU system as described on Fig 4. An inspection of the results obtained with the reference system on the trials on day one where athletes performed the same sub-technique during the whole trial was used to validate the decision tree.

## Results

In total, 32’958 ski cycle and 45’319 poling cycles were detected by the reference system, and more than 99% of the temporal events were detected by the IMU method. Table 1 shows the number of events detected with the IMU compared to the reference system, within each sub-technique. The number of cycles with correct sub-technique determination is also provided in Table 1.

**Table 1:** Number of events detected by the infrared camera reference system (REF), and the percentage of events (%) detected by the inertial measurement units (IMU), as well as the number of cycles sub-technique correctly assessed.

	G2		G3		G4		All Gear	
	REF	%	REF	%	REF	%	REF	%
Pole initial contact	9489	97.2	25381	99.9	10449	99.3	45319	99.2
Pole final contact	9489	98.2	25381	99.9	10449	99.6	45319	99.5
Ski initial contact	9655	99.1	12750	97.4	10553	98.4	32958	98.2
Ski final contact	9655	99.2	12750	99.2	10553	99.4	32958	99.2
Sub-technique	9571	94.4	12421	98.5	10379	97.1	32371	96.8

Pole and ski initial and final contact events detection errors, as well as the corresponding inner-cycle phases duration errors, are presented in Table 2. Positive differences indicate that the event was detected earlier on the IMU data than on the reference. The b_μ_ ranged between -49 and +30 ms across the different sub-techniques with a b_σ_ between 7 and 74. The highest precision σ_μ_ was reached by the P_ON_ with 7 ms error, followed by the S_OFF_ with 14 ms errors, while the P_OFF_ and S_ON_ reached 30 ms and 56 ms respectively.

**Table 2:** Time differences between the temporal events determined using a reference system (multiple infrared cameras) and using inertial measurement units (IMUs), as well as for the inner-cycle parameters.

	G2				G3				G4				All Gear			
	b_μ_	b_σ_	σ_μ_	σ_σ_	b_μ_	b_σ_	σ_μ_	σ_σ_	b_μ_	b_σ_	σ_μ_	σ_σ_	b_μ_	b_σ_	σ_μ_	σ_σ_
Pole initial contact	-24	23	6	13	-24	16	7	5	-27	17	4	3	-22	17	7	6
Pole final contact	-5	33	13	35	-16	19	16	16	-15	23	7	8	-10	22	15	21
Ski initial contact	3	27	30	30	1	31	54	25	18	24	33	24	9	24	45	24
Ski final contact	3	25	9	4	-8	19	17	12	-2	29	13	9	-1	24	14	10
Pole contact time	19	29	23	57	9	15	18	18	10	13	9	11	12	15	19	27
Pole swing time	-12	33	37	79	-4	16	18	18	-5	14	10	15	-7	15	22	35
Ski contact time	-4	15	33	30	-9	38	63	39	-25	27	37	27	-9	26	50	29
Ski swing time	2	16	39	40	8	41	63	31	21	23	37	46	8	25	54	31
Ski cycle time	0	4	46	42	-1	6	75	44	-1	9	47	50	0	4	66	40
Pole contact time %	3.1	4.7	3.6	9.7	2.4	4.1	5.0	4.8	2.9	4.0	2.5	2.9	2.8	3.5	4.5	6.3
Pole swing time %	-1.8	4.3	5.2	10.3	-0.7	2.4	2.8	2.8	-0.4	1.1	0.9	1.6	-0.8	2.2	3.0	4.7
Ski contact time %	-0.5	2.1	3.9	4.6	-1.0	3.2	5.1	3.3	-2.7	3.0	3.9	3.0	-1.1	2.7	5.1	3.3
Ski swing time %	0.4	3.1	6.9	7.6	0.8	5.4	7.9	4.5	2.9	3.5	5.4	6.0	1.1	3.5	7.8	4.9
Ski cycle time %	0.0	0.3	3.5	3.9	-0.1	0.3	3.8	2.3	-0.1	0.5	3.0	2.9	-0.1	0.3	3.8	2.2

Time differences are expressed in milliseconds (ms) and in percentage of the inner-cycle phase duration. “b” and “σ” are the abbreviations for bias (intra-trial mean error) and precision (intra-trial standard deviation of the error), respectively, while subscript “μ” and “σ” represent the median and the interquartile range over all the trials.

A central feature for the analysis of the inner-cycle parameters is σ_μ_, describing the precision of the phase calculation across the different trials. In absolute terms, the temporal parameters associated to the poles were more precise than the one calculated for the skis, with a range from 9 to 33 ms for the P_CT, P_SW, and a range from 33 to 75 ms for S_CT, S_SW and S_CY. When calculating the errors relatively to the corresponding phase duration, the temporal parameters associated with the poles achieved a precision range between 0.9% and 5.2%, while the relative errors associated with the skis ranged between 3.0% and 7.9%.

**Fig 5:**
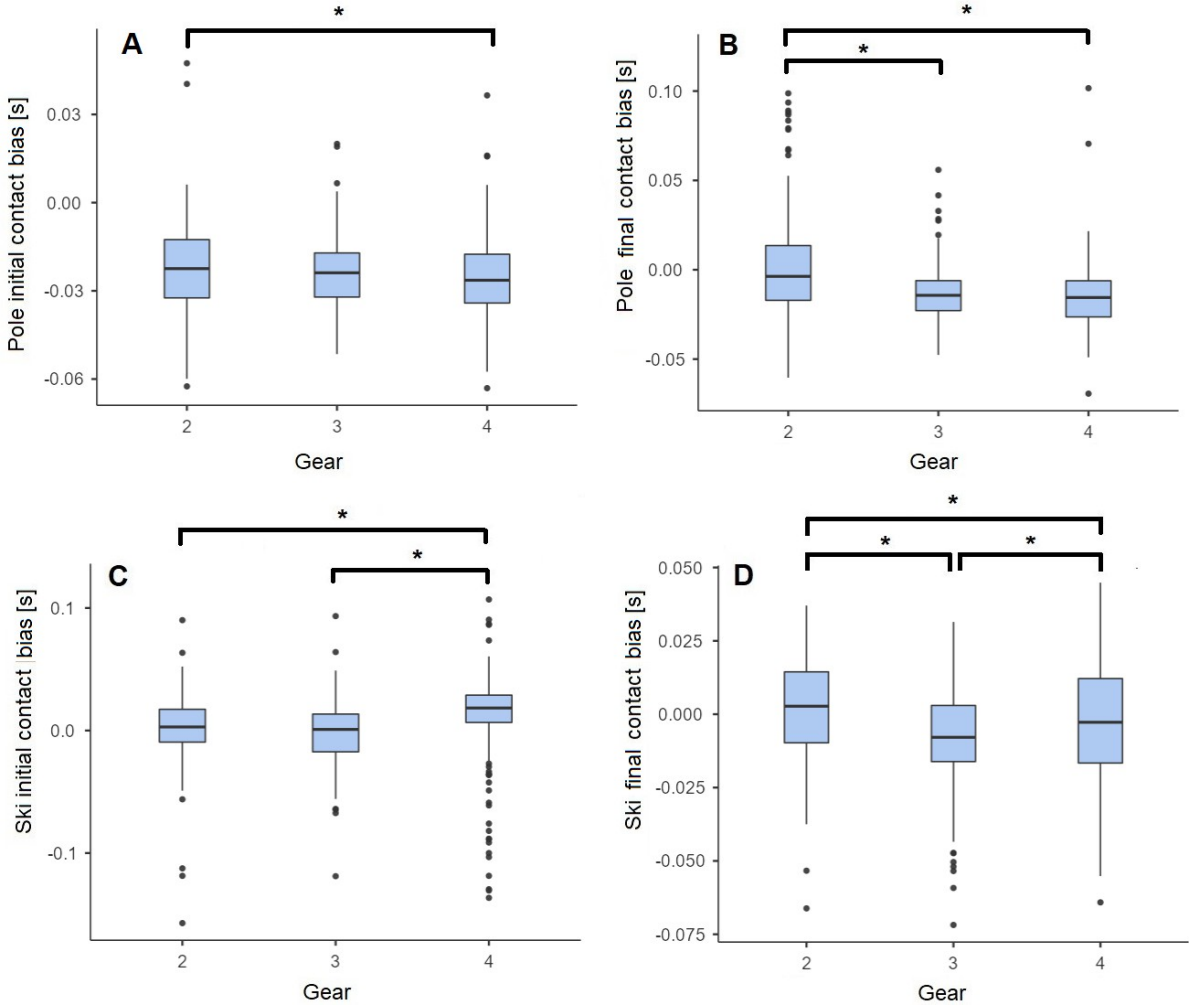
**A:** Pole initial contact (P_ON_), **B:** Pole final contact (P_OFF_), **C:** Ski initial contact (S_ON_), **D:** Ski final contact (S_OFF_) inter-trials bias for Gear 2 (G2), Gear 3 (G3) and Gear 4 (G4) sub-techniques. * indicates significant pairwise differences based on an alpha level of < 5%.

The influence of sub-technique on the b_σ_ of the temporal events is presented in Fig 5. There is a significant difference in the assessment of the temporal events (e.g., P_ON,_ P_OFF,_ S_ON_ and S_OFF_) depending on the used sub-technique. For the poles, P_ON_ is detected later with the IMUs system in G4 compared to G2, while P_OFF_ is detected later in G3 and G4 compared to G2. For the skis, S_ON_ happen a bit earlier with the IMU method for G4 compared to G2 and G3, while S_OFF_ is earlier in G2, a bit too late in G4 and even later in G3.

**Fig 6:**
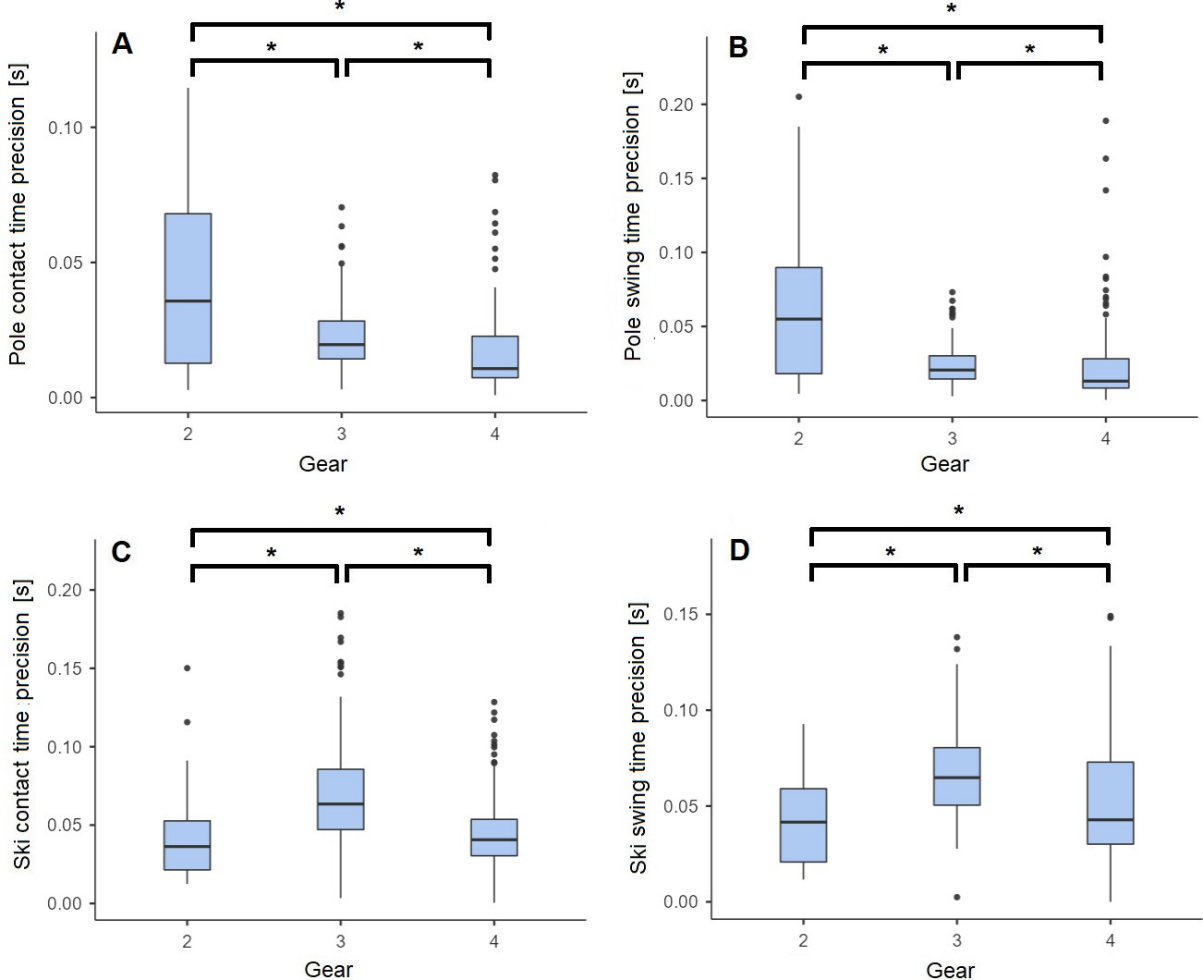
**A:** Pole contact time (P_CT), **B:** Pole swing time (P_SW), **C:** Ski contact time (S_CT), **D:** Ski swing time (S_SW) inter-trials precision for Gear 2 (G2), Gear 3 (G3) and Gear 4 (G4) sub-techniques. * indicates significant pairwise differences based on an alpha level of < 5%.

For the deduced temporal parameters (e.g., P_CT, P_SW, S_CT, S_SW and S_CY) the influence of the sub-technique on the σ_μ_ of the temporal parameters is presented in Fig 6. There is a significant difference in the assessment of the temporal parameters between each sub-technique (all p<0.001). P_CT and P_SW have a lower precision in G2, while S_CT and S_SW have lower precision in G3.

## Discussion

The present study aims to validate the use of temporal events of poles and skis from IMUs to determine inner-cycle parameters within the main skating sub-techniques in roller skiing. Here we were able to successfully detect more than 99% of the events with the IMUs. Corresponding inner-cycle parameters were determined with a precision between 9 ms and 75 ms for the different phases, representing a relative error from 0.9% to 7.9% of the corresponding inner-cycle duration.

When comparing the detected temporal events, P_ON_ was detected with the best precision (4 ms for G4 to 7 ms for G3) compared to the other events, as the peak acceleration for an IMU placed on the wrist is clearly visible in the acceleration signal. This precision reaches the resolution on the reference system. The P_OFF_ was a bit more difficult to detect and showed a slightly worse precision (7 ms for G4 to 16 ms for G3). The motion of the arms at the end of the poling phase usually depends on the sub-technique and a variation of the motion pattern between the athletes. Some athletes will have a small break after the P_OFF_ before the repositioning phase of the poles, while other will immediately reposition the poles to start a new cycle. A varying delay can therefore be expected for this parameter, compared to the reference system that truly measure when the pole leaves the treadmill. A similar behaviour was highlighted by Fasel et al. [15] for the precision of the pole’s events in the classical style, with better precision for the initial contact than for the final contact. For the skis, S_OFF_ was sometimes difficult to assess based on the vertical acceleration following the ski contact phase, as this vertical motion is not always sharp. A good precision between 9 ms for G2 and 17 ms for G3 was achieved. Finally, the S_ON_ was the most difficult event to assess, reaching precision between 30 ms for G2 and 54 ms for G3. The ground contact of the two wheels can occur either simultaneously or one may follow the other with a time delay, affecting the determination of the event. This lower precision in the determination of S_ON_ was also observed by Myklebust [21]. Overall the precision obtained for the different events were of the same order of magnitude as previously found in classical [15], and skating [21] styles, but a bit less precise than in running [12].

For the calculated inner-phases temporal parameters, the precision for both absolute and relative values were better for the parameters deduced from the poles than from the skis. The precision around 20 ms for P_CT and P_SW is slightly better than previously published work in classical [15, 27] and skating [21] styles. For the temporal parameters related to the skis, the precision cannot be compared to results obtained for the thrust phase in classical style, as the skis stay motionless on the ground for a short period of time. This feature is easily recognised on the IMU data and allows for an precise (4 ms) estimation of the ski thrust phase [15]. It was therefore expected to obtain less precise results. The S_CT and S_SW obtained in our study could more adequately be compared to the ski gliding and recovery duration in classic, with also slightly better precision obtained in the present study (50–54 ms vs 65–66 ms) [15]. The S_CY, calculated using the S_ON,_ provide the worse precision due to the difficulty to assess the initial contact of the wheels on the ground. Using the S_OFF_ or the P_ON_ to define cycles could clearly improve the precision of this parameter.

In term of relative error, the different temporal parameters provided precision between 3.0% and 7.8%, also varying depending on the sub-technique. Such precision would likely allow detecting of possible differences between skiers of different performance levels. For example, athletes of international and national level have showed differences above 10% in cycle rate when roller skiing at similar speeds [28]. It was also shown in a previous study that a similar IMUs configuration could be used to highlight technique differences and training intervention [20]. When looking at other disciplines, it has been shown that fatigue protocols could affect temporal parameters in the magnitude of 5% between pre and post fatigue exercise [29, 30]. It could therefore be estimated that the method provided here has the sensibility to detect evolution of the temporal parameters due to fatigue, at least for cycle duration, pole contact time, and pole swing time.

The precision of the inner-cycle temporal parameters could probably be further improved by increasing the resolution of the reference system. The temporal resolution of the 3D camera system (i.e., 5 ms) could be increased, and the method used to determine poles and skis contact to the ground could lead to slightly different outcome that with the IMUs, mainly for the detection of S_ON_ and P_OFF_ as seen previously. While running on an instrumented treadmill equipped with force plate recording at 1000 Hz, Falbriard et al. [12] reached a precision ranging between 3 and 5 ms for inner-cycle phases duration. This kind of precision could probably also be reached in cross country skiing. The location of the IMUs could also affect the precision of the detection. The choice of the wrists and skis to attach the IMUs was made based on previous work to offer the best solution for both data quality and a possible place for a commercial device [22, 23]. Having the IMU on the pole could possibly provide slightly better precision but the advantage compared to having a validation for a possible use of a smart watch was considered as low. However, an interesting temporal parameter that cannot be determined using such a method is the free gliding phase of the skis, when the skier doesn’t push with the legs or arms to provide energy [31]. There is indeed no temporal event correlated to this free gliding phase that can be detected from the IMU data.

The determination of the sub-technique based on a decision tree and temporal events provided good results. The percentage of correct classification is directly linked to the quality of the events detection. The results obtained are similar to the ones obtained in the field with machine learning in classical style [32] and using a decision tree in roller ski skating [33]. In both studies, the main issue was to detect the turns, but in our case, such a situation was not a concern as movements were only performed straight-forward on the treadmill.

When comparing b_μ_ for P_ON_, P_OFF_, S_ON_ and S_OFF_, we found significant differences between sub-techniques. Thus, some improvements would be expected by taking the sub-technique into account to correct the bias. Moreover, as the synchronisation between the reference system and the IMUs was performed manually, a systematic error may have been introduced. Finally, the precision of the temporal parameters was also affected by the sub-technique. The second gear showed higher variability than G3 and G4 for the P_CT and P_SW. This can be explained by the asymmetry of the arms motion in G2 [21, 31], as the left and right poles events are merged and by the reduction of cycle time and the fast arm motion on steep slopes, where G2 is used.

## Conclusions

This study showed the validity of using IMUs on athlete’s wrists and skis to determine temporal events, inner-cycle parameters and the performed sub-technique in treadmill roller ski skating. With precision ranging between 19 ms and 66 ms, corresponding to 3.0% to 7.8% of the corresponding inner-cycle duration, this precision would likely allow differentiation of skiers on different performance levels. Overall, this laboratory validation provides interesting possibilities also for outdoor use.
